# Photocatalytic Hydrogen Evolution of TiZrNbHfTaO_x_ High-Entropy Oxide Synthesized by Mechano-Thermal Method

**DOI:** 10.3390/ma17040853

**Published:** 2024-02-11

**Authors:** Ömer Güler, Mustafa Boyrazlı, Muhammet Gökhan Albayrak, Seval Hale Güler, Tatsumi Ishihara, Kaveh Edalati

**Affiliations:** 1 Rare Earth Elements Application and Research Center, Munzur University, Tunceli 62000, Turkey; sevalhaleguler@munzur.edu.tr; 2 Metallurgical and Materials Engineering Department, Engineering Faculty, Fırat University, Elazig 23119, Turkey; mboyrazli@firat.edu.tr (M.B.); mgalbayrak@firat.edu.tr (M.G.A.); 3 WPI, International Institute for Carbon-Neutral Energy Research (WPI-I2CNER), Kyushu University, Fukuoka 819-0395, Japan; ishihara@cstf.kyushu-u.ac.jp

**Keywords:** high-entropy alloys, high-entropy oxides, photocatalysis, mechano-thermal process, hydrogen production

## Abstract

One of the most promising solutions to slow down CO_2_ emissions is the use of photocatalysis to produce hydrogen as a clean fuel. However, the efficiency of the photocatalysts is not at the desired level, and they usually need precious metal co-catalysts for reactions. In this study, to achieve efficient photocatalytic hydrogen production, a high-entropy oxide was synthesized by a mechano-thermal method. The synthesized high-entropy oxide had a bandgap of 2.45 eV, which coincided with both UV and visible light regions. The material could successfully produce hydrogen from water under light, but the main difference to conventional photocatalysts was that the photocatalysis proceeded without a co-catalyst addition. Hydrogen production increased with increasing time, and at the end of the 3 h period, 134.76 µmol/m^2^ h of hydrogen was produced. These findings not only introduce a new method for producing high-entropy photocatalysts but also confirm the high potential of high-entropy photocatalysts for hydrogen production without the need for precious metal co-catalysts.

## 1. Introduction

Intensive human activities over the last century has caused an unprecedented impact on the Earth’s climate. With the industrial revolution, carbon dioxide (CO_2_) emissions began to increase. Due to the increase in population and energy consumption since the Second World War, CO_2_ emissions continue to increase every year [1]. It is known that today, CO_2_ emissions are over 33 billion tons [2]. According to the Paris climate agreement that was signed in 2015, there is an aim not to exceed 10 billion tons of emissions annually until 2050 in order to limit the temperature increase to 2 °C. However, the increase in global energy demand continues to increase every year. In the face of such a difficult situation, the development of carbon-neutral energy sources has become more important than ever [3].

From past to present, some known technologies for converting solar energy into electrical energy, heat energy, and chemical energy have been reported. In addition, photocatalytic hydrogen production from water has attracted intense interest since 1972, when it was reported that some semiconductors act as catalysts in the photocatalytic splitting of water [4,5,6]. Producing hydrogen from water photocatalytically has some advantages. For example, water is the most abundant clean resource on Earth. In addition, hydrogen that is obtained as a result of the splitting of water is an environmentally friendly fuel. Hydrogen itself is a clean fuel, and when it is burned, it releases water and energy. Considering these advantages, such a fuel cycle will solve the important problems that humanity is currently experiencing. However, in order to use such a fuel in our daily lives, highly effective catalysts that can split water under sunlight are needed [7,8,9,10,11,12]. There are some factors that affect efficiency in energy production. Light absorption, electron-hole separation and transport, and the photoelectron chemical reaction are the most important factors [13]. Therefore, the effectiveness of a photocatalyst is the key to producing H_2_ by water splitting. Among the heterogeneous semiconductor catalysts that are commonly used in photocatalytic hydrogen production, a wide variety of catalysts such as TiO_2_ [14,15,16], CdS [17], BiVO_4_ [18], Ta_3_N_5_ [19], gC_3_N_4_ [20,21], and high-entropy ceramics (HECs) [22] have been used.

The concept of high-entropy materials was first introduced by Yeh et al. in 2004 [23]. They said that an alloy that is obtained by mixing five or more metals in equimolar or near-equimolar amounts will be an alloy with a high mixing entropy. Numerous studies on this subject since then have revealed that these alloys have a single- and/or two-phase structure and exhibit many superior properties compared to traditional alloys. Studies have shown that the concept of high entropy is valid not only in alloys but also in ceramics, as high-performance ceramic materials can be obtained with the concept of high entropy. HECs, consisting of five or more cations, are a new generation of materials that exhibit superior properties compared to traditional ceramics [22]. The Gibbs free energy of these ceramics is low because their entropy is high, which gives high-entropy alloys (HEAs) high stability under different conditions, including catalytic reactions [22]. The presence of at least five cations with different atomic sizes in HECs causes natural lattice stress and defects to occur [24]. Moreover, HECs exhibit unprecedented properties in the field of catalysis due to four important features: high entropy, hysteresis diffusion, lattice distortion, and the cocktail effect [25,26]. The most popular types of HECs are high-entropy oxides (HEOs), high-entropy nitrides, high-entropy carbides, and high-entropy borides. Due to their superior properties, they can replace traditional ceramics. Beyond the classic use of HECs, there are few studies on their use as photocatalysts [27,28,29].

The first study on the photocatalytic properties of HECs was carried out by Edalati et al. [29]. In that study, a powder mixture consisting of Ti, Zr, Nb, Hf, and Ta metals was converted into a HEA by the high-pressure torsion (HPT) method, and then, TiZrNbHfTaO_x_ HEO was obtained by oxidation of the HEA at high temperatures. Despite the potential of the HPT method in discovering new materials, the amount of produced samples from this method remains small. Moreover, because of the nature of HPT, in which the strain is proportional to the distance from the rotation center, it is hard to achieve a homogenous mixture of elements without using a very large number of turns. It is expected that using new methods to achieve homogenous mixing of elements at the atomic scale can result in better performance of high-entropy photocatalysts, such as higher efficiency or activity without co-catalyst addition.

In this study, a new synthesis route was applied to produce high-entropy photocatalysts. A TiZrNbHfTa HEA was produced by the mechanical alloying method. Then, the obtained HEA was oxidized by a mechano-thermal method to obtain TiZrNbHfTaO_x_ HEO. The synthesized alloy could produce hydrogen from water without the need to use a co-catalyst, which is an advantage compared to synthesis by the high-pressure method.

## 2. Materials and Methods

Ti (Merck, Darmstadt, Germany, 99%), Zr (Nanografi, Ankara, Turkey, 99.5%), Nb (Sigma-Aldrich, Burlington, MA, USA, 99.8%), Hf (Alfa Aesar, Haverhill, MA, USA, 99.6%), and Ta (Alfa Aesar, Haverhill, MA, USA, 99.9%) powders were weighed and mixed equimolarly. They were exposed to an MA process (for 120 h) in a high-energy ball milling machine (Retsch PM 100 brand, Haan, Germany). The MA process was carried out at 400 rpm in an argon atmosphere using 12 mm diameter hardened steel balls. At the beginning and at the end of the mechanical alloying process, the powders were subjected to X-ray diffraction (XRD, Rigaku with Cu Kα radiation, Tokyo, Japan), scanning electron microscopy (SEM, Hitachi SU3500 (Tokyo, Japan) with an acceleration voltage of 15 kV), and energy-dispersive X-ray spectroscopy (EDS, Oxford AZtech, High Wycombe, UK) examinations.

**Figure 1:**
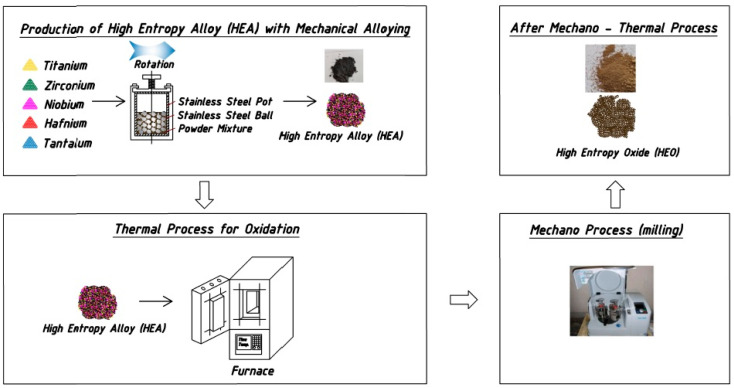
Schematic representation of the production process of HEO.

The mechano-thermal method was used to convert HEA powders obtained by mechanical alloying into an HEO. This process was carried out in two stages. In the first stage, HEA powders were oxidized in an air atmosphere for 12 h in an oven at 1100 °C, and then, the obtained oxide powders were ground in a high-energy ball milling machine (Retsch PM 100, Haan, Germany) at 400 rpm for 5 h using steel balls with 12 mm diameter. In the second stage, the ball-milled oxide powders were oxidized again in the oven at 1100 °C in the air atmosphere for 12 h and then ground for 5 h by ball milling in the same way as the first stage. A total of 24 h of oxidation and 10 h of grinding were carried out. The obtained powders were subjected to XRD, SEM, and EDS analyses. Additionally, they were exposed to X-ray photoelectron spectroscopy (XPS, Specs-Flex, Berlin, Germany) analysis using Al K_α_ radiation to characterize the oxides that were formed. The production process of the samples is shown schematically in Figure 1.

The photocatalytic activity of samples was examined by hydrogen evolution in water using a 300 W Xe lamp. For photocatalysis, 50 mg of HEO was dispersed in 27 mL of H_2_O, with 3 mL of methanol (as a sacrificial agent). The photocatalytic test was performed for several cycles, and the production of oxygen and hydrogen gases was quantified using a gas chromatograph equipped with a thermal conductivity detector. The specific surface area of the photocatalyst was determined by Brunauer–Emmett–Teller (BET) analysis [24].

## 3. Results

**Figure 2:**
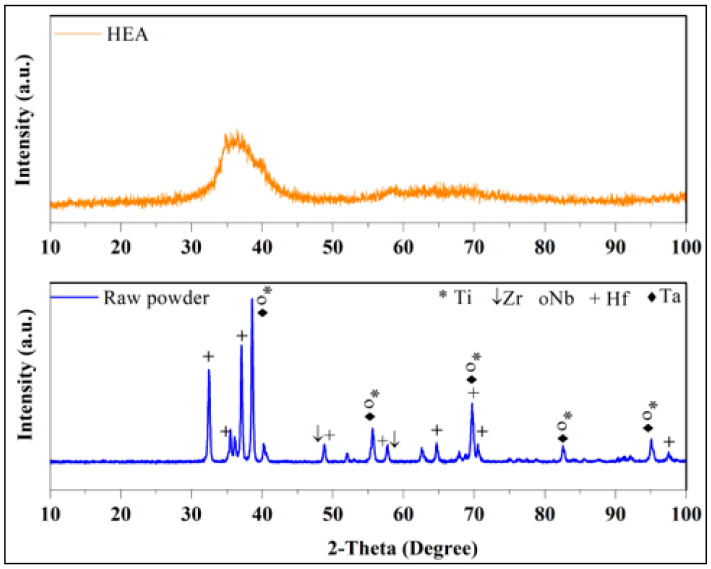
XRD spectra of TiZrNbHfTa HEA obtained before and after mechanical alloying of the powder mixture consisting of Ti, Zr, Nb, Hf, and Ta.

The XRD spectrum of the HEA, which was obtained as a result of mechanical alloying, is given in Figure 2. The XRD spectrum of the starting powder mixture is also shown in Figure 2 for comparison. Characteristic peaks of Zr, Ta, Nb, Hf, and Ti metals are seen in the XRD spectrum of the initial powder mixture. After 120 h of mechanical grinding, the peaks disappear, and an amorphous structure emerges. This is expected due to the nature of mechanical alloying. During mechanical alloying, under the influence of ball impacts, metal powders are first cold-welded, and then broken and cold-welded again. Moreover, the micro-strain increases in metal powders by severe plastic deformation due to the effect of ball impacts [30]. Micro-strain occurring in metal powders causes the crystal size to decrease and the crystal structure to deteriorate. At the end of mechanical alloying, the structure becomes completely amorphous [31,32].

**Figure 3:**
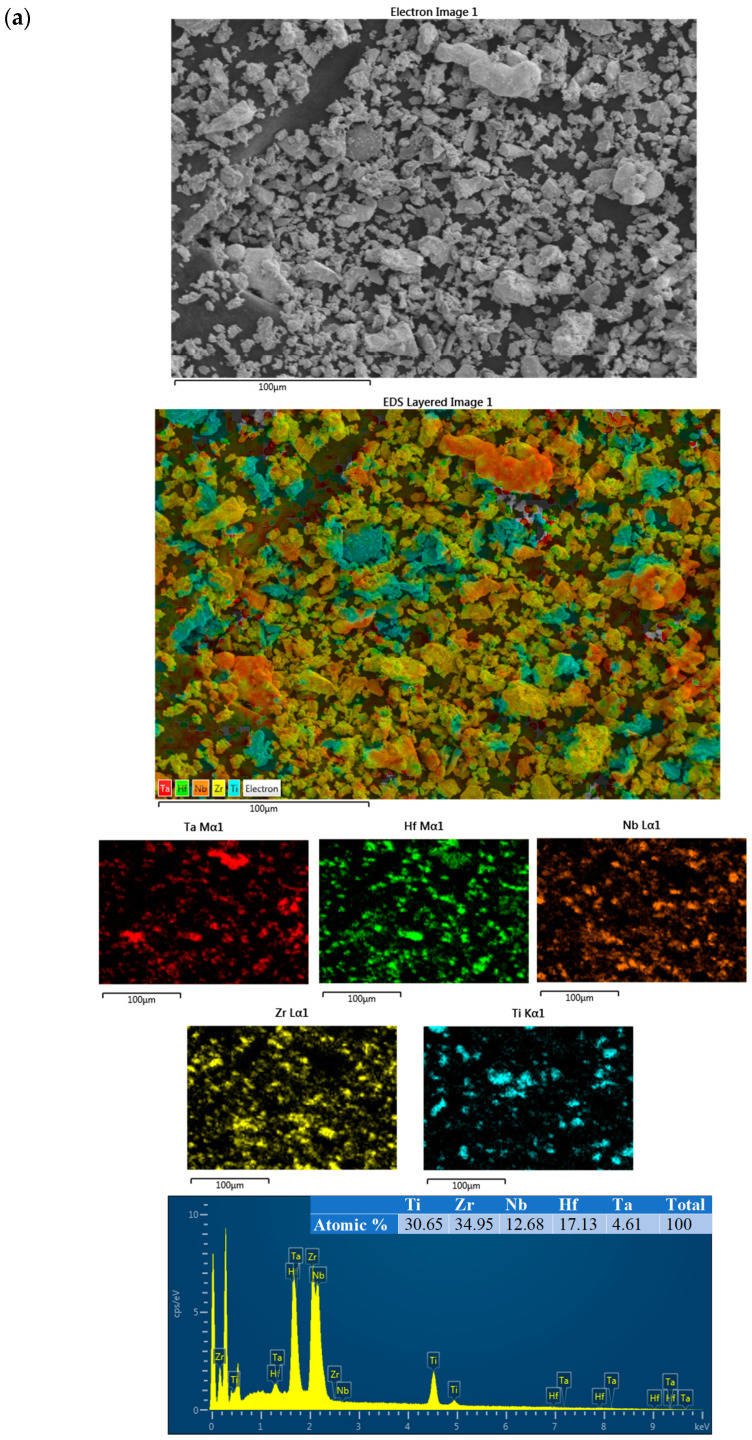

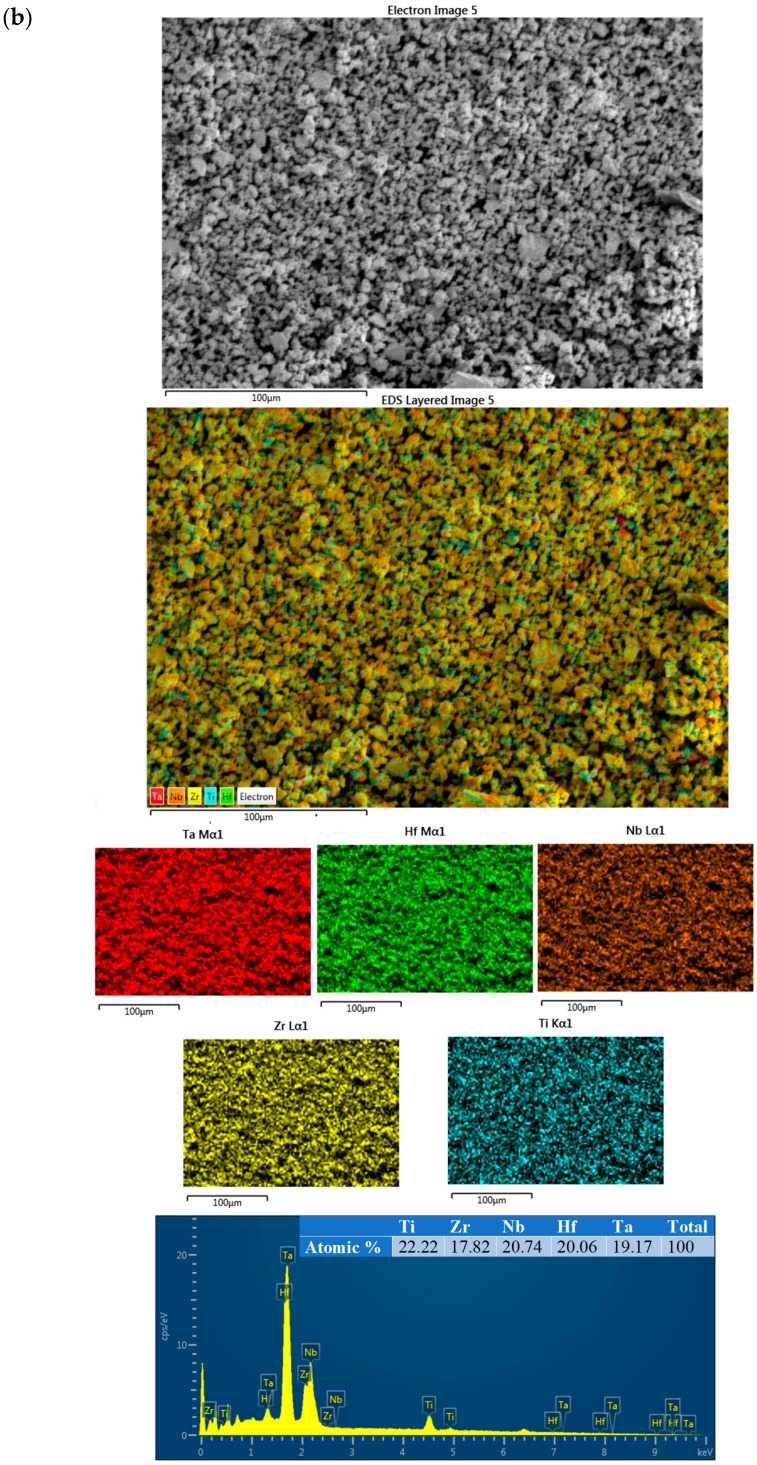
(**a**) SEM-EDS analysis of the powder mixture consisting of Ti, Zr, Nb, Hf, and Ta before mechanical alloying; (**b**) SEM-EDS analysis of TiZrNbHfTa HEA obtained after mechanical alloying.

Figure 3 shows the SEM-EDS analysis results of the TiZrNbHfTa HEA (a) before and (b) after mechanical alloying for 120 h. Figure 3a shows that the initial powder mixture consists of Ti, Zr, Nb, H, and Ta elements before mechanical alloying. As can be seen from the figure, the powder mixture has different particle shapes and different particle sizes, and there is no homogeneous distribution in the powders. Figure 3b shows that the particles after mechanical alloying are spherical in shape and are close in size to each other. Moreover, the distribution of elements is quite homogenous after mechanical alloying.

A two-stage mechano-thermal process was applied to convert TiZrNbHfTa HEA powders into TiZrNbHfTaO_x_ HEO. In previous studies, TiZrNbHfTa HEA powders were oxidized for 24 h [28,33]. It should be noted that oxides of Ti, Zr, Nb, Hf, and Ta metals are refractory, and their formation as a protective oxide layer on the surface may hinder the oxide from going further inside [34,35,36,37,38]. Therefore, directly oxidizing an HEA that is composed of these metals may cause the surface to oxidize and the core to remain unoxidized. Therefore, in this study, the HEA alloy was first oxidized for 12 h and then ground to break up the oxide films and was oxidized again in an attempt to fully oxidize the alloy.

**Figure 4:**
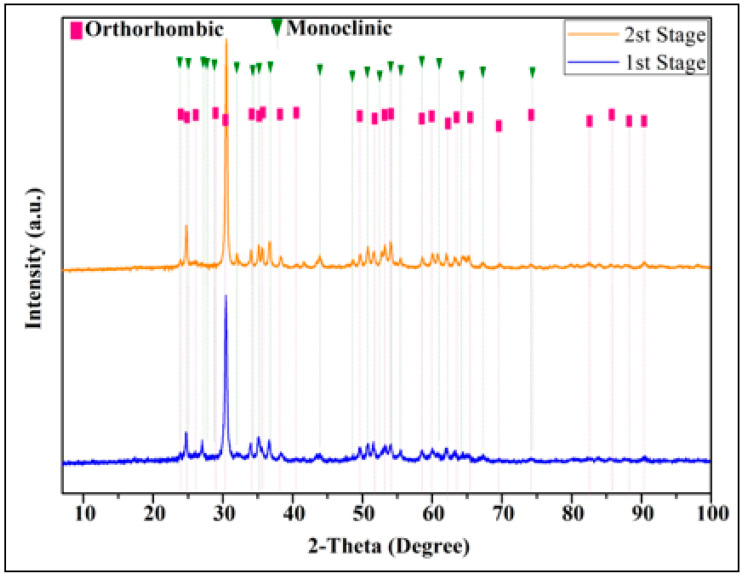
XRD spectrum of HEO obtained by oxidation of HEA by mechano-thermal method.

The XRD spectra are given in Figure 4 to show the changes that occur after the first and second stages of oxidation. As can be seen from the figure, oxides were formed after the first stage of the mechano-thermal process. After the second stage of the process, an increase in the intensity of the peaks was observed. It is thought that the reason for this situation is that the unoxidized amorphous phase, which remained after the first stage, was oxidized in the second stage. The final product contains two phases of monoclinic (JCPDF card number: 01-085-7298) with an A2/m space group and orthorhombic (JCPDF card number: 01-087-7051) with an Ima2 space group, which is in good agreement with earlier publications [24,29]. Some peaks and their miller indices are as follows: for the monoclinic phase, 23.94° (110), 24.59° (−111), 26.0° (003), 26.31° (−602), 26.89° (−311), 27.07° (−601), 28.09° (−112), 31.16° (112), 32.46° (−511), 31.69° (−313), 32.78° (402), 33.63° (−113), 35.79° (601), 38.95° (511), 44.03° (005), 47.85° (−1012), 55.18° (−1113), 59.34° (514), 60.95° (−822), and 67.04° (025); for the orthorhombic phase, 30.28° (811), 33.93° (002), 35.05° (1600), 36.30° (020), 38.34° (802), 49.58° (1602), 50.54° (022), 58.47° (813), 61.62° (831), and 67.23° (1613).

**Figure 5:**
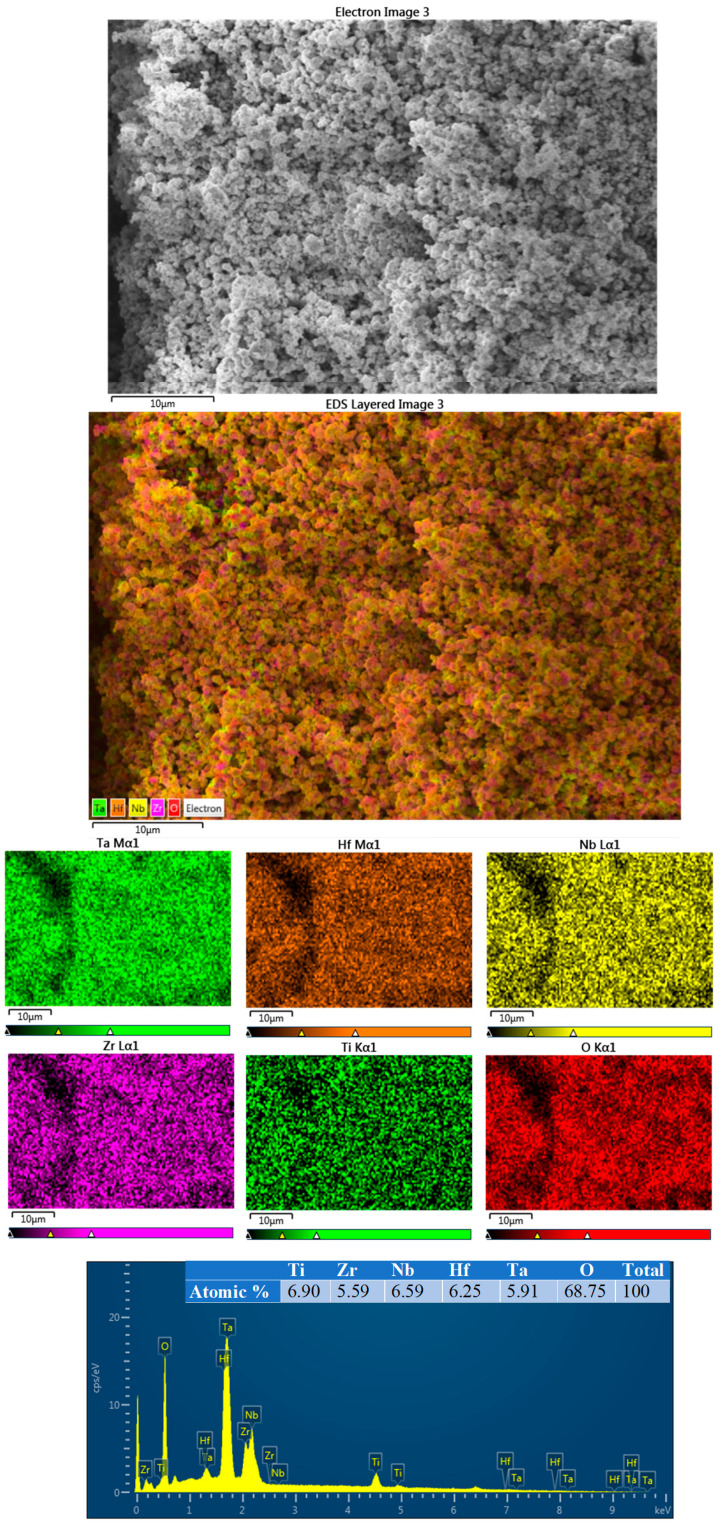
SEM-EDS analysis of HEO obtained by oxidation of HEA by mechano-thermal method.

Figure 5 shows the SEM-EDS analysis of the HEO that was obtained as a result of the mechano-thermal process. HEO particles are a few micrometers in size. In addition, the distributions of both metals and oxygen in HEO are very homogeneous.

**Figure 6:**
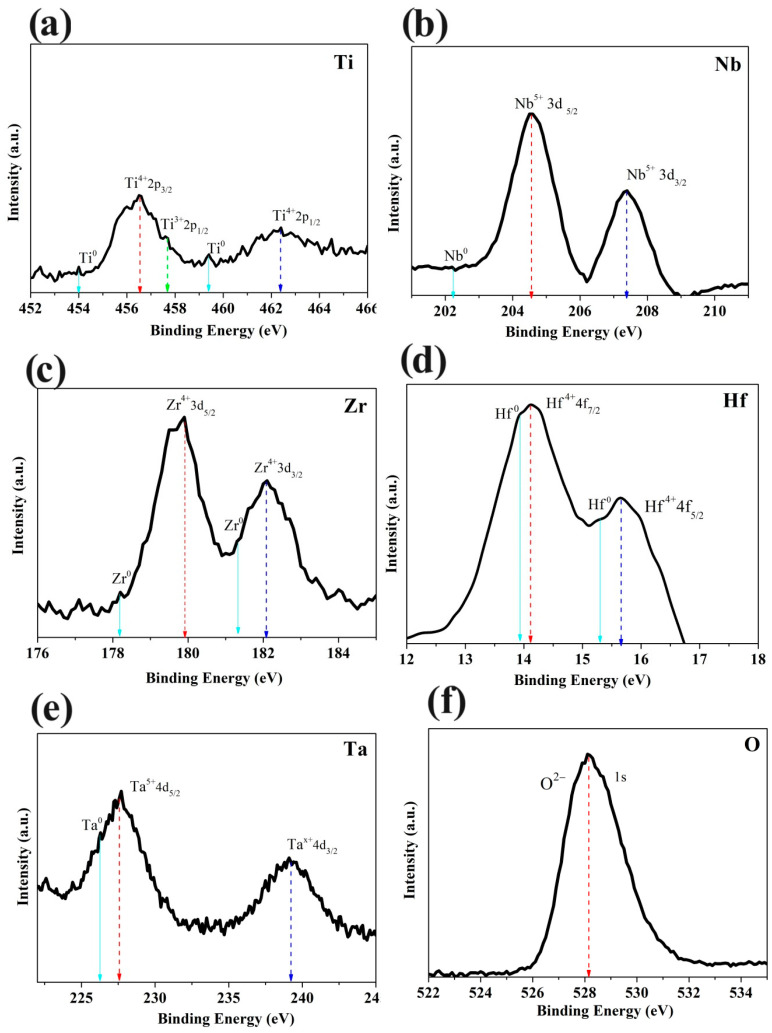
XPS analysis for (**a**) Ti, (**b**) Nb, (**c**) Zr, (**d**) Hf, (**e**) Ta, and (**f**) O in HEO TiZrHfNbTaO_x_.

In order to determine the success of HEO production with the mechano-thermal method, XPS analysis was performed on the samples and further analyzed by peak deconvolution, as shown in Figure 6. The main oxidation states of cations in the sample are Ti^4+,^ Zr^4+^, Nb^5+^, Hf^4+^, and Ta^5+^, indicating that the material is successfully oxidized to a d^0^ electronic configuration during the two-stage mechano-thermal oxidation.

**Figure 7:**
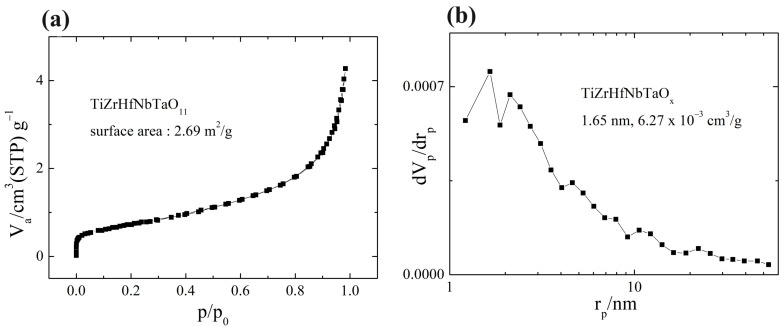
(**a**) BET and (**b**) pore size distribution of HEO (STP: standard temperature and pressure, p/p_o_: relative pressure, V: absorbed volume, r_p_: pore diameter, and dV_p_/dr_p_: pore volume).

The pore characteristics of samples were examined with low-temperature nitrogen absorption and desorption measurements. The surface area of HEO was found to be 2.69 m^2^/g. As seen in Figure 7a, the HEO exhibits a typical isotherm combining the characteristics of type I, characterized by a sharp uptake in the low-pressure region (micropore). The analysis of pore sizes in HEO is given in Figure 7b. The average pore size is 1.65 nm.

**Figure 8:**
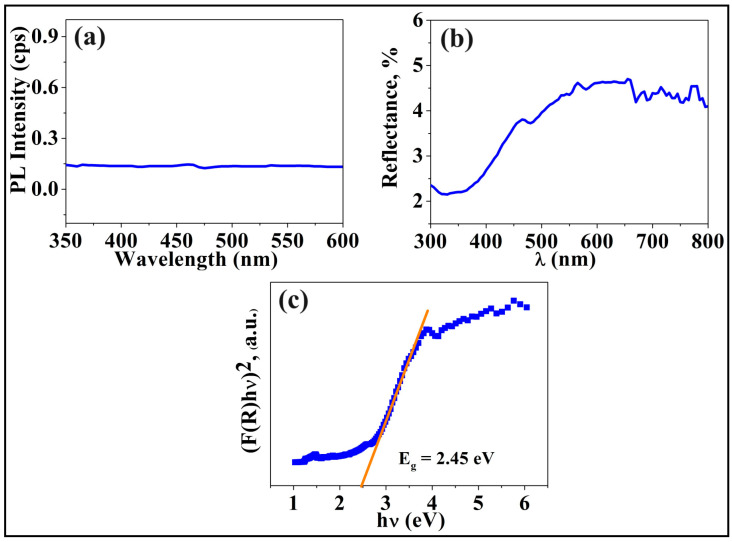
(**a**) Photoluminescence spectrum for HEO, (**b**) UV-vis light reflectance spectrum, and (**c**) Kubelka–Munk plot to calculate the bandgap.

By photoluminescence spectroscopy, the behavior of photoexcited charge carriers was examined, and this is shown in Figure 8a. No clear photoluminescence peaks appear for the sample, suggesting that the recombination of electrons and holes to produce photoluminescence is not so significant. Therefore, electrons have a longer time to contribute to photocurrent generation and photocatalysis.

The investigations of the light reflectance of TiZrHfNbTaO_x_ with UV-vis spectroscopy (Figure 8b) and Kubelka–Munk analysis (Figure 8c) show some important points. The sample absorbs most of the incoming light and reflects a small part of it, as shown in Figure 8b. In addition, the prediction of the bandgap by using the Kubelka–Munk analysis, as shown in Figure 8c, demonstrates that the bandgap of the material is 2.45 eV, which is within the visible light region.

**Figure 9:**
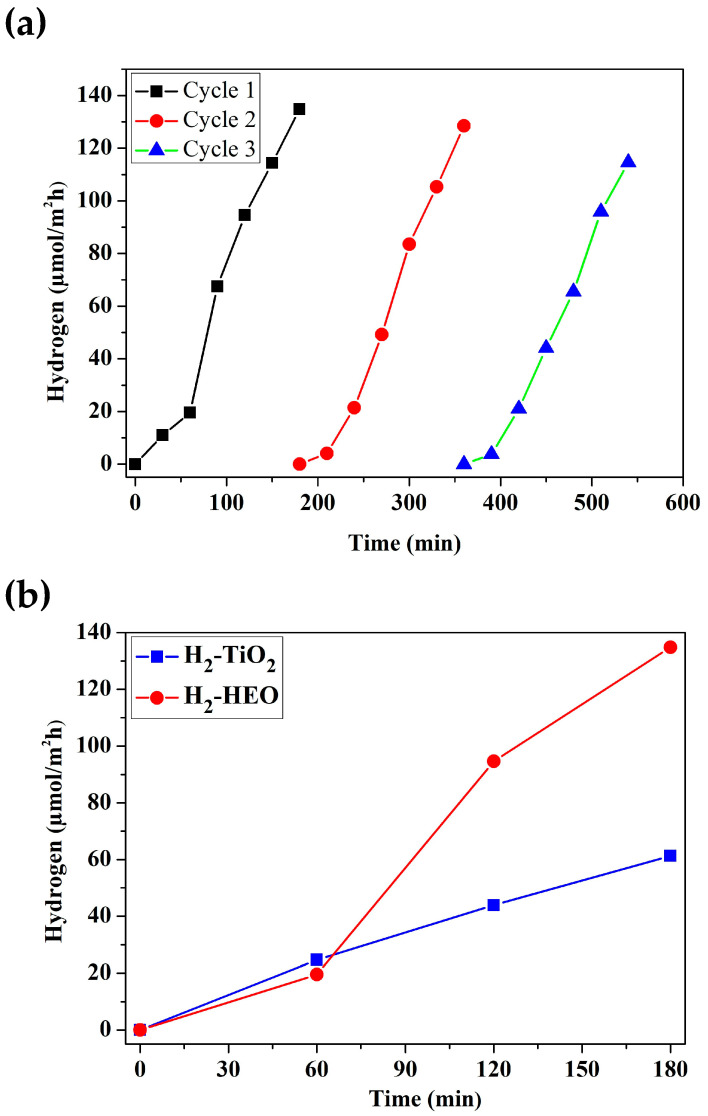
(**a**) Cyclic photocatalysis on HEO TiZrHfNbTaO_x_ and variation of produced hydrogen against exposure time using Xe UV source for three cycles. (**b**) Comparison of photocatalytic hydrogen production on HEO TiZrHfNbTaO_x_ and anatase TiO_2_ (**a**,**b**).

Although the band gap of the TiZrNbHfTaO_x_ high-entropy oxide is 2.45 eV, it does not produce a measurable amount of hydrogen under visible light within the detection limit of the gas chromatograph. Therefore, photocatalytic water-splitting experiments were performed with an Xe lamp. Photocatalytic water splitting was carried out under UV light, as shown in Figure 9a, in order to examine the photocatalytic activity of the TiZrHfNbTaO_x_. The amount of produced hydrogen increases linearly with increasing exposure time. It was confirmed that the synthesized HEO can act as a photocatalyst for hydrogen production without co-catalyst addition. Figure 9a shows that the amount of photocatalytic hydrogen production is reasonably similar for the three cycles, demonstrating the long-term stability of the photocatalyst.

After 30 min photocatalysis in the first cycle, the hydrogen production increased to 11.02 µmol/m^2^ h, while 180 min later, it was 134.76 µmol/m^2^ h. Although the increase in the period was 6 times, the increase in the produced product was more than 12 times. In the second cycle and the third cycle, the production of hydrogen after 30 min decreased to 7 µmol/m^2^ h compared to the first cycle. However, in the second and third cycles, the hydrogen production after 180 min was 128.43 µmol/m^2^ h and 114.58 µmol/m^2^ h, respectively. There was a slight decrease in hydrogen production in the second and third cycles. The small reduction in activity during cycling is probably due to the deactivation of some active sites, as observed in many other photocatalysts.

The most known catalyst for water splitting is the traditional TiO_2_ photocatalyst. In this study, in order to reveal the efficiency of the HEO catalyst, that was produced by the mechano-thermal method, the TiO_2_ (anatase) catalyst was also tested under the same experimental conditions. As can be seen from Figure 9b, TiO_2_ could also produce hydrogen. Although the BET surface area of the commercial TiO_2_ that was used was higher (10.2 m^2^/g), its hydrogen production was lower than the hydrogen production of the HEO. Both TiO_2_ and HEO catalysts produced hydrogen at similar values in the first 60 min of hydrogen production, but a significant increase in the hydrogen production of the HEO was observed after 60 min.

## 4. Discussion

In this study, the TiZrHfNbTaO_x_ catalyst (with an average particle size of around 1–3 µm) was obtained by the mechano-thermal method and exhibited photocatalytic behavior that was similar to the one previously produced by Edalati et al. [29] using a high-pressure method. The amounts of hydrogen that were produced and the long-term photocatalytic activity in this study are similar to those reported in ref. [29]. However, an important difference was detected. While a co-catalyst such as Pt is needed in the catalyst that was produced by Edalati et al., the catalyst that was produced by the mechano-thermal method can produce hydrogen without a co-catalyst. Such a difference can be attributed to a more uniform distribution of elements in this study and the fully oxidized states of cations, which could be achieved by a two-stage mechano-thermal process. Hydrogen production without a co-catalyst addition can rarely be achieved in conventional photocatalysts [39,40,41], and thus, this study confirms another advantage of high-entropy photocatalysts for hydrogen production. This feature can be attributed to the presence of various elements with different distributions of electrons which can produce appropriate sites for donating and accepting electrons for photocatalytic reaction. Future studies using theoretical calculations are needed to clarify the mechanism of the good activity of high-entropy photocatalysts without co-catalyst addition.

In Table 1, the amounts of hydrogen that were obtained by water splitting in the literature using conventional photocatalysts are given and compared with the current high-entropy photocatalyst. The highest hydrogen production belongs to the Au-CdS catalyst, with 472.26 µmol/h m^2^. The hydrogen production of the catalyst used in this study is almost 12 times lower (44.85 µmol/h m^2^). Although the bandgap values of these two compared catalysts are very close to each other, there is a 3-fold difference between their BET surface areas. Moreover, it should also be noted that most of the conventional catalysts that are used and reported in Table 1 require a co-catalyst, while the HEO catalyst used in this study produced hydrogen without the need for a co-catalyst.

**Figure 10:**
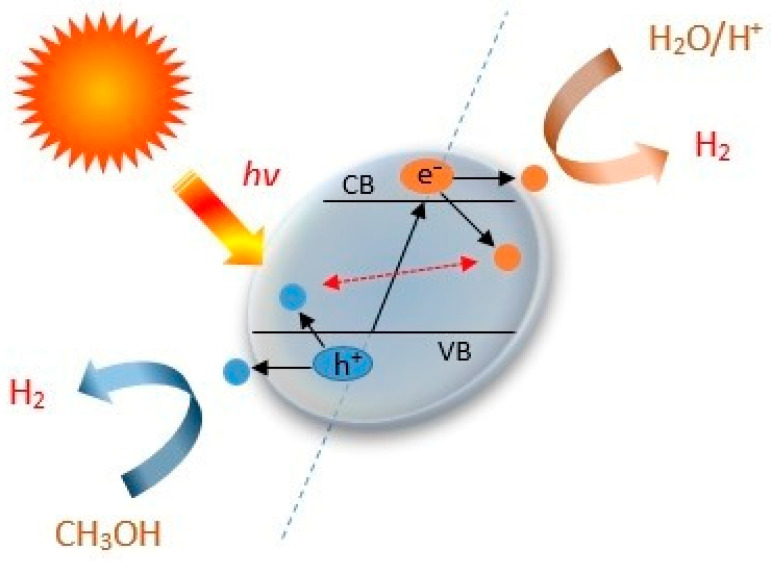
Schematic representation of hydrogen production mechanism of photocatalyst HEO.

The photocatalytic H_2_ production process of HEO is shown schematically in Figure 10. Redox reactions take place on a photocatalyst after electron-hole (e^−^/h^+^) pairs are generated under light irradiation with energy that is higher than the bandgap (E_g_) of the semiconductor.

In this study, the reaction occurring during the water-splitting process is as follows [43]:

HEO + UV light → e^−^ + h^+^ (1)

H_2_O + h^+^ → OH^•^ + H^+^ (2)

H^+^ + e^−^ → OH^•^ + 1/2H_2_ (3)

OH^•^ + CH_3_OH → CO_2_ + 3/2H_2_ or OH^•^ + CH_3_OH → HCHO + H_2_O +1/2H_2_ (4)

In this study, conduction band electrons become more readily available to reduce electron-acceptor species, e.g., protons that produce hydrogen. The used methanol is able to combine with photogenerated valance band holes and hydroxyl radicals and act as a sacrificial agent in the photocatalytic production of hydrogen. An overall water splitting did not occur on this HEO photocatalyst, and holes were consumed from methanol, which is a well-known sacrificial agent [24].

The HEO that was produced in this study is TiZrNbHfTaO_x_. As mentioned before, the band gap of the HEO is 2.45 eV. TiO_2_, ZrO_2_, HfO_2_, Nb_2_O_5_, and Ta_2_O_5_ have bandgaps in the range of 3.1–5.7 eV, with negligible light absorbance in the visible light region. Therefore, the absorbance of the produced HEO under visible light is better than the absorbance of single or binary oxides. This shows that HEOs have high potential as catalysts in photocatalytic water splitting [24,29].

A high mixing entropy of these materials reduces the free internal energy of the materials and improves their stability under thermal or chemical conditions, which is a benefit for photocatalysis. In addition, the presence of several cations with hybridized orbitals can control the valence electron concentration and consequently tailor the electronic structure for a particular property. It is well known that the cations with the d_0_ and d_10_ electronic configurations exhibit good photocatalytic activity, and this fact was used in this study to select the composition of the HEO [24,29].

In addition to improved photocatalytic activity, the method used in this study has the potential to produce much larger amounts of samples compared to the high-pressure method that was used earlier by Edalati et al. [29]. Such powders have other merits such as a large surface area, which is three times higher than those achieved by the high-pressure method. Moreover, the structure that was achieved in this study is porous, which provides another advantage for photocatalysis such as mesoporous catalysts [44,45,46,47]. It is also worth mentioning that the simplicity of the process in this method makes it appropriate for discovering new photocatalysts for other reactions as well.

## 5. Conclusions

In this study, a two-stage mechano-thermal method was used for the first time to produce photocatalysts for hydrogen production from water splitting. The synthesized TiZrNbHfTaO_x_ high-entropy oxide had a mesoporous structure with a reasonable surface area and could produce hydrogen by photocatalysis without co-catalyst addition. The current results not only show the importance of the synthesis method in the photocatalytic performance but also introduce high-entropy ceramics as active photocatalysts without the need for precious metal co-catalysts.

## Figures and Tables

**Table 1:** Photocatalysts used for water splitting in the literature and their hydrogen production amounts [42].

Catalyst Name	E_g_ (eV)	BET Surface Area (m^2^/g)	Result (µmol/m^2^ h)
Ag_2_O/TiO_2_	2.86	24.65	135.90
Au–TiO_2_	2.76	77.14	93.34
GO–TiO_2_	2.58	42.72	88.95
TiO_2_–ZnO	3.06	85.91	15.02
CdS	2.40	24.57	48.84
Au–CdS	2.40	8.47	472.26
CdS/Ti	2.63	138.41	0.34
Bi_2_S_3_/Pt/ZnO	2.71	27.45	0.84
ZnO/ZnS	3.40	34.67	11.20
In_2_O_3_/Ta_2_O_5_	2.80	43.75	12.18
Rh/Cr_2_O_3_/GaZn	2.60	48.30	30.81

## Data Availability

Data are contained within the article.
